# Examining a novel firefighter exercise training program on simulated fire ground test performance, cardiorespiratory endurance, and strength: a pilot investigation

**DOI:** 10.1186/s12995-019-0232-2

**Published:** 2019-04-23

**Authors:** Brittany S. Hollerbach, Sara A. Jahnke, Walker S. C. Poston, Craig A. Harms, Katie M. Heinrich

**Affiliations:** 1 Department of Kinesiology, Kansas State University, Natatorium 8, 920 Denison Ave., Manhattan, Kansas, 66506 USA; 2 Center for Fire, Rescue, and EMS Health Research, National Development & Research Institutes, 1920 143rd Street, Suite 120, Leawood, KS 66224-7813 USA

**Keywords:** Firefighter, Health, Occupational health, Fitness, HIFT

## Abstract

**Background:**

Firefighting is a dangerous occupation with high rates of injuries and fatalities, with the majority of line of duty fatalities due to cardiovascular events. Additionally, firefighters struggle with poor health/low levels of fitness, including high (> 80%) rates of overweight and obesity. Limited resources exist for fire departments that are tailored to the culture and work requirements of these “tactical athletes”. Though there has been increasing interest in high intensity functional training (HIFT) programs, research data are lacking among firefighters and few studies have focused on training recruits. The purpose of this pilot investigation was to examine a novel HIFT program (TF20) on fire academy recruits’ health, fitness, and performance as determined by a simulated fire ground test (SFGT), as well as determining the program’s acceptability and feasibility.

**Methods:**

Thirteen participants were recruited from an entry level fire academy and were randomly assigned to the control (CG, *n* = 6) or HIFT group (TF20, *n* = 7). The CG was asked to continue current exercise habits. TF20 was provided a 10-week online based training program that included periodized workouts, nutritional information, and mental readiness education. Due to attrition within the first two weeks of the study, 10 male fire recruits (23 ± 3 years) completed the study (CG, *n* = 3, TF20, *n* = 7). All 10 participants completed baseline and follow-up assessments.

**Results:**

The TF20 group showed improvement on numerous outcome measures including SFGT (40% passing at baseline, 86% passing post-intervention). TF20 participants significantly increased estimated VO2max (*p* = 0.028), improved body composition (p = 0.028), and improved grip strength (*p* = 0.018). The CG did not experience any significant changes. The TF20 group completed approximately 75% of the assigned workouts.

**Conclusion:**

While TF20 participants showed significant fitness gains, the small sample size limited direct comparisons to the CG. TF20 was well-received although there may be a better way to implement the intervention to increase participation. This investigation provides promising outcomes, useful information about implementation, feasibility, and acceptability for the TF20 HIFT program among firefighter recruits. IRB #8063 APPROVED 01/04/2016.

**Trial registration:**

NCT03319394. Registered 28 September 2014. Retrospectively registered.

## Background

Firefighting is a strenuous and physically demanding occupation [1]. Firefighters (FFs) work in dangerous and complex environments, which increases their risk for injuries and fatalities [2]. In addition to the dangerous nature of the job, FFs struggle with poor health and low levels of physical fitness, including very high (> 80%) rates of overweight and obesity (body mass index [BMI] ≥ 25.0 kg/m^2^), likely related to the culture of the fire service [3]. Many firefighters experience significant weight gain over the course of an approximate 25-year career, with a range of 29–85 pounds gained (i.e., 1.15–3.4 lbs./year; 3). As a firefighter’s weight increases, their cardiorespiratory fitness plummets and their risk of cardiovascular disease (CVD) increases [4]. Comorbidities related to overweight and obesity include heart disease, stroke, type 2 diabetes and certain types of cancer which are highly prevalent among the firefighter population [3, 5].

It is well documented that physical fitness is related to job performance, including the performance of simulated firefighting tasks that are relevant to actual job tasks (e.g., pulling hose, carrying a ladder, and rescuing a victim; 3). Firefighting presents a unique challenge for physical fitness training because it requires concurrently improving multiple fitness training goals [6]. Firefighting requires optimal levels of power, strength, muscular endurance, and anaerobic/aerobic endurance [6, 7]. Inadequate fitness levels may reduce the occupational performance and increase the risk of overexertion injuries for firefighters [7]. On the other hand, increased physical fitness is related to lower levels of injury/illness, reduced absenteeism, increased productivity, and increased work capacity for firefighters [6].

Limited resources exist for fire departments that are tailored to the culture and work requirements of these tactical athletes [6]. There has been increasing interest in high-intensity functional training (HIFT) programs among tactical populations, yet key research data are lacking for the firefighter population [6, 8]. The National Fire Protection Association (NFPA) has several standards that focus on the health risks of firefighters (e.g., NFPA 1500, NFPA 1582, and NFPA 1583; [9–12]). While these guidelines exist, there are no nationally-enforced fitness or physical activity requirements for firefighters, which leads to inconsistent fitness training within and between fire departments, substandard fitness levels, and greater risks for obesity, injury, and cardiovascular-related events [9]. For example, only 38.7% of career and 23.6% of volunteer firefighters meet the fitness threshold suggested as a minimal return to work post cardiac event by NFPA 1582 which includes a VO_2max_ of at least 42 ml/kg/min [9, 11].

The NFPA recommends firefighters be allowed to exercise on duty to maintain adequate fitness levels [12]. While Poplin et al. (2012) found that on-duty physical exercise was responsible for one-third of all firefighter injuries (32.9%), most of these injuries tended to be minor strains/sprains [13]. It is still recommended that firefighters be encouraged to exercise while on duty. Studies have shown that firefighters who engaged in regular physical training were less likely to incur a serious injury on the fireground [14, 15]. Furthermore, firefighters who train regularly and possess higher fitness levels tend to perform job-specific tasks more efficiently than untrained firefighters, emphasizing the importance of implementing an exercise program for firefighters [7, 13, 16]. Thus, there is a need for cost-effective training programs targeted at the unique needs and culture of the fire service as a means of improving readiness, decreasing injury, and preventing line of duty deaths (LODD) related to CVD.

Fire academies provide instruction for new firefighters and should instill the importance of physical fitness training as recruits begin a physically demanding career in the fire service [17]. However, current fire academies across the U.S. do little to address physical fitness other than the physical skills taught during fire training [17]. The applied coursework requires much time spent on the drill ground learning basic firefighter skills, e.g., donning personal protective equipment such as bunker gear and self-contained breathing apparatus (SCBA), navigating through dark search quarters, searching and removing a victim, climbing ladders, and fighting live fire. Firefighter recruits also are typically required to take the Candidate Physical Ability Test (CPAT), or an equivalent occupational fire ground test during the Fire Academy, which they must pass to be considered for employment in most fire departments across the country [18]. These tests are physically demanding and require high fitness levels.

The purpose of this pilot investigation was to assess the acceptability, feasibility, and relevant fitness and occupational performance outcomes of an innovative firefighter HIFT program, The First Twenty Tactical High Performance Program (TF20) with firefighter recruits. This is the first investigation to examine TF20 among firefighter recruits and significantly adds to the existing literature regarding firefighter fitness training. We hypothesized that the intervention group (TF20) would have greater improvements in performance, fitness, and health outcomes, as described below.

## Methods

The aims of this pilot study were to examine the acceptability, feasibility, and fitness and occupational performance outcomes among firefighter recruits after a 10-week randomized trial. The institutional review board (IRB) of Kansas State University (KSU) approved the study (IRB #8063). All participants provided written consent prior to initiation of their involvement in research.

### Participants

We solicited study volunteers by visiting a midwestern fire academy that is hosted by a community college fire science degree program with permission from the Fire Academy Program Director. Participants (*N* = 13; 92.3% male) reported they were in good health and without physical limitations that prevented them from completing any of the required TF20 workouts and fitness assessments.

Participants were individually randomly assigned to either the intervention (TF20) or comparison group (CG). Microsoft Excel was used to generate the random allocation sequence. Both groups completed baseline assessments, 10-weeks of either the TF20 or CG, and follow-up assessments.

**Fig. 1:**
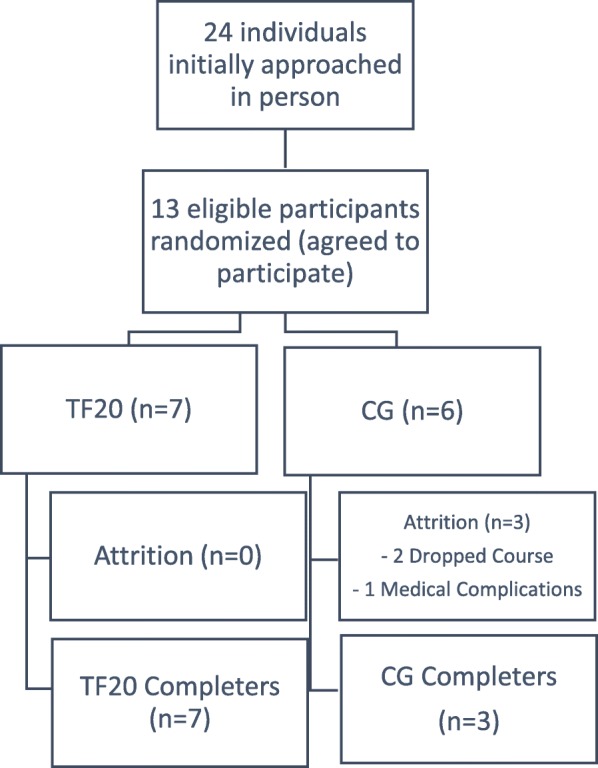
Randomization of Eligible Participants. Legend: TF20 = The First Twenty intervention; CG = comparison group

Because recruits also were students in the community college degree program, passing the CPAT prior to entry into the fire academy was not required. The fire academy provided instruction to prepare recruits to take state tests for firefighter certification, but they were not professional firefighters. Figure 1 shows participant randomization and progress through the study (TF20 = 7 and control [CG] = 6).

### Intervention

TF20 is an innovative online training program developed by firefighters specifically for firefighters that provides foundational educational principles around physical fitness, mental wellness, and nutrition. Using a holistic approach combined with empirical evidence [19–21] TF20 is a comprehensive program that addresses firefighters’ unique physiological challenges by simulating tasks performed on the fire ground. Specifically, the program’s goals are to optimize a firefighter’s occupational performance, resilience to injury, stability, mobility, strength, and endurance through a series of high intensity circuits, focused on both resistance and endurance exercises. TF20 online platform allows participants to apply and track these principles on a daily basis and record their progress. The portal includes private account settings, health programs, fitness tracking with exercise and workout videos, nutrition tracking, health education, results tracking, and communication and social media tools.

TF20 Intervention Group (TF20) workouts were part of an online training program that provided endurance and resistance exercises, nutritional information, and mental performance guidance. The original program was a 24-week periodized program which was condensed into a 10-week exercise program to accommodate the time frame of the study and fire academy academic semester. A National Strength and Conditioning Association (NSCA) Certified Strength and Conditioning Specialist (CSCS) and Tactical Strength and Conditioning Facilitator (TSAC-F) condensed the program but kept the periodization scheme consistent to mimic the longer cycle of workouts. The workouts are summarized in Table 1.

**Table 1:** The first twenty (TF20) study periodization cycle

Week	Mesocycle	Exercise description	Time/duration (days/week, d/wk)
1	1	Resistance Training	2 d/wk
2		Moderate-Intensity Cardiovascular Training	2 d/wk. in 20 min. bouts
3	2	Resistance Training	2 d/wk
4		Moderate-Intensity Cardiovascular Training	2 d/wk. in 30 min. bouts
5		Walk with SCBA	1 d/wk. for 20 min.
6	3	Resistance Training	2 d/wk
7		Moderate Cardio	2 d/wk. in 45 min. bouts
8		Vigorous Cardio (stair climb w/SCBA)	1 d/wk. for 14 min
9	4	Resistance Training	2–3 d/wk
		Moderate Intensity Cardio	1 d/wk. in 60 min bouts
		Vigorous Cardio (walk w/SCBA)	1 d/wk. in 20–30 min bouts
10		Active Recovery	

Workouts contained a combination of aerobic (e.g., running, rowing, jumping), body weight (e.g., air squats, pushups, sit-ups), and weight lifting (e.g., presses, back squats, weighted lunges) exercises with workouts designed to use equipment available in an exercise facility (e.g., weight racks, benches) or in a fire station/on the fire ground (e.g., equipment carry, dummy drag, etc.). Sixty-minute TF20 sessions included a warm-up, workout, and cool down. All completed workouts were logged in TF20 software program, which was used to assess intervention adherence.

The comparison group (CG; i.e.: control group) followed their regular workout routine for 10 weeks. Participants chose the time, duration, frequency, and type of workouts completed. For the study, participants were asked to log all exercise/workouts online using Google Sheets.

Both the TF20 and CG met weekly with a firefighter fitness trainer with current CPR and First Aid certifications (Firefighter/EMT, CPR/First Aid, TSAC-F certified). Weekly meetings ensured education on proper movements used in TF20 program, and discussion of proper movement progressions. Both groups were reminded to log their workouts. Any questions the participants had were also discussed during the meetings.

### Procedures

The research team enrolled participants during the first week of their 16-week fire academy. The research team generated the random allocation and assigned participants to intervention groups. Baseline testing occurred during the second week of the fire academy, the intervention took place the next 10 weeks and the follow-up assessments were completed within the following two weeks.

As an incentive to participants, this study took the place of the one credit hour physical fitness course requirement for the fire academy. This one credit hour course was offered through the local community college and allowed participants to access the community college gym. The class was not associated with the fire academy nor was it instructor-led; it solely provided access to a gym. The fire academy in this study did not have a structured exercise (fitness training) component.

### Measures

During the initial (baseline) and final 10-week (follow-up) of the study, participants’ health, fitness, and performance were assessed. A Seca stadiometer (Chino, CA) was used to assess height. The Tanita TBF-300A digital bioelectrical impedance analysis (BIA) scale (Arlington Heights, IL) was used to assess body composition including percent body fat (%BF), fat mass (FM in kg), fat-free mass (i.e., muscle mass; FFM in kg), body mass index (BMI in kg/m^2^), and weight (to the nearest 0.1 kg). Research has shown that BIA correlates well (r > 0.8) with the gold-standard measure dual X-ray absorptiometry (DEXA) analysis for body composition [22].

Maximal aerobic capacity (VO_2max_) was estimated from the linear relationship between heart rate (HR) and work rate during a submaximal graded exercise test using a StairMaster StepMill 7000PT [23, 24]. A standardized protocol recommended for firefighters by the Joint Labor Management Wellness Fitness Initiative (WFI) was used [23]. This test is recommended for firefighters because, unlike other submaximal graded exercise tests, this one requires the participant to exercise to maximal volitional fatigue, similar to completing a physically exhausting task on the fireground [23, 24]. Additionally, studies using this test and prediction equation demonstrated accuracy and validity, with no false positives or negatives [24].

Upper body muscular strength (grip strength; GS) was assessed with the Takei 5401 Hand Grip Dynamometer (Digital; Niigata City, Japan). A hand grip dynamometer is a valid and reliable measure (*p* < 0.05) and is a strong correlate (r > 0.9994) with upper body muscular strength [25, 26]. Participants’ dominant hand was noted; GS was recorded three times for both hands in an alternating fashion. The best of three attempts was recorded for each hand. GS was examined alone (unadjusted) and in relation to the participant’s body weight (adjusted).

Upper body muscular endurance was assessed with a 2-min timed push-up (PU) test [27]. The PU test required participants to touch their chin to the mat at the bottom of each repetition, and the score was the number of continuous repetitions completed [27].

Muscular power was assessed with a counter-movement jump (Jump, Sunnyvale, CA). The best of three jumps was recorded. Participants’ standing reach height was subtracted from their maximal jump height, so their total vertical displacement was recorded to the nearest cm.

Core strength was assessed with a cadence curl-up test [27]. Two strips of masking tape were placed 12 cm apart. Participants lay supine across the tape with knees bent 90°. A metronome was set at 40 beats/min. At each beep the participant curled their body upwards so as to move their hands to the second tape line. Repetitions were counted each time the participant reached the bottom position. The test was concluded either when the participant completed 75 curl-ups or the cadence was broken [27].

Agility was assessed with a timed agility T test [28]. This assessment required participants to move in a T-shaped pattern requiring lateral and front-to-back movements. The agility test was recorded in seconds to completion.

Trunk flexibility was assessed using a standardized sit-and-reach box (Canadian Trunk Forward Flexion test; 27).

A simulated fire ground test (SFGT), the Candidate Physical Ability Test (CPAT) was used to measure occupational performance, fitness, and agility. The CPAT provides a traditional frame of reference to evaluate increases or decreases in physical fitness and occupational readiness and provides a firefighter-specific assessment. The CPAT consists of eight separate events that require the participant to progress along a predetermined path from event to event in a continuous manner. It is a pass/fail test based on finishing all events in a maximum total test time of 10 min and 20 s or less. Participants were provided with a familiarization session prior to taking the CPAT for the first time as is suggested by the IAFF/IAFC [29]. Participants were able to familiarize themselves with the equipment at each event but did not take the entire test during the familiarization period.

In all eight events (i.e., Stair Climb, Hose Drag, Equipment Carry, Ladder Raise and Extension, Forcible Entry, Search, Dummy Drag, and Ceiling Breach and Pull), candidates wore a 50-pound (22.68-kg) vest to simulate the weight of self-contained breathing apparatus (SCBA) and firefighter protective clothing. An additional 25 pounds (11.34 kg), using two 12.5-pound (5.67-kg) shoulder weights that simulated a high-rise pack (hose bundle), was added for the stair climb event. Throughout all events, candidates wore long pants, a hard hat with chin strap, work gloves and footwear with no open heel or toe. Watches and loose or restrictive jewelry were not permitted. All props were designed to simulate critical fire ground tasks and test the candidate’s physical ability [29]. Participants’ heart rate and blood pressure were taken immediately (within two minutes) following completion of the CPAT.

A questionnaire was completed at baseline and follow-up and included standard demographics (baseline only), health behaviors, current exercise habits, and current nutritional habits. Physical activity was measured using the modified International Physical Activity Questionnaire (IPAQ) short form, which provided a global, physical activity self-rating during the last 30 days [30]. Participants were asked to indicate the amount of moderate and vigorous aerobic activity and strength training completed. From this, researchers created a dichotomous variable for meeting/not meeting the physical activity guidelines for the previous 30 days. Participants were also asked if they followed any current diet/meal plans and if their diet had changed in the previous 12 weeks.

A feasibility analysis was completed to examine participant adherence, their reactions to the intervention, and suggestions for future physical exercise training for the Fire Academy.

### Statistical analysis

Microsoft Excel and SPSS Version 21 (Armonk, NY) were used for statistical analyses. Means, standard deviations, and proportions were calculated for all variables. However, the small sample size and uneven groups precluded the use of typical parametric between-groups comparisons. In order to examine outcomes of all participants initially recruited in the study, we used an Intention to Treat (ITT) Model carrying forward the baseline observations for those that did not have post-test values. This allowed us to examine all participants, assuming no change for those that did not complete the intervention. The Mann-Whitney U Test was used to examine differences between the two groups. Within-group changes over time for both groups were examined for the completers using the Wilcoxon Signed Rank Test to compare repeated measures (pre- and post-intervention) for each group separately. The Wilcoxon converts scores to ranks and compares them at Time 1 (pre-) and Time 2 (post-) [31]. Statistical significance was set at *p* < 0.05.

Written responses to questionnaires (baseline and follow-up) were analyzed qualitatively by coding and analyzing recurrent themes, areas of consensus and convergence of opinions, experiences, and perceptions about the wellness program using a grounded theory approach [32]. Data were then coded by identifying passages exemplifying key concepts or ideas related to the major themes using NVivo 10 (QSR International, 2016). A feasibility analysis also was completed to examine the relevance of offering this intervention in a fire academy. Adherence to the prescribed workouts for TF20 group and their feedback to the intervention were examined in a follow-up questionnaire*.*

## Results

Thirteen participants consented to study participation (TF20: *n* = 7, 100% male, 22.6 ± 2.9 years; CG: *n* = 6, 83% male, 23.5 ± 3.6 years). Participant demographic characteristics are presented in Table 2. The Mann-Whitney U test revealed there were no significant differences at baseline between the two groups with respect to demographic factors or on pre-intervention fitness measures. Ten male fire service recruits (aged 19–27 years) completed the study, including baseline and follow-up assessments and 10 weeks of either the TF20 intervention or self-guided exercise (TF20 n = 7, CG *n* = 3). Overall, the participants were less physically active than expected at baseline. Of the nine (6 TF20, 3 CG) that filled out a baseline questionnaire regarding current exercise behavior (over the past 30 days), 44% of TF20 group (*n* = 4) and 67% of the CG (*n* = 2) met either moderate or vigorous aerobic PAG. Only four (all in TF20 group) met the current physical activity guidelines (aerobic and muscle-strengthening).

**Table 2:** Participant demographics at baseline (N = 13)

Variable	CG	TF20	Difference
Age (years)	23.5	22.6	−0.9
Gender (%Male)	83.0	100.0	+ 17.0
Weight (kg)	72.4	90.0	+ 17.6
^a^BMI (kg/m^2^)	23.9	29.2	+ 5.3
VO_2max_ (ml/kg/min)	42.3	38.5	−3.8

^a^Body Mass Index: underweight (> 18.5); normal weight (18.5–24.9); overweight (25–29.9) and obese (≥30)

### Dropouts

One participant (CG) dropped out of the Fire Academy only two weeks into data collection and was therefore ineligible to complete the study. One participant (CG) dropped out of the study due to time constraints during the 10-week intervention and another participant (CG) completed the 10 weeks of self-directed workouts but was unable to complete follow-up testing due to diabetes-related medical complications. All three of the subjects who dropped out of the study were assigned to the CG, accounting for the subsequent uneven group distribution.

### Between group differences on fitness measures

Using the ITT model, we compared differences between groups. The Mann-Whitney U test revealed the two groups differed significantly in two measures. The TF20 group had significantly greater improvements in grip strength and the CG had significantly greater reductions in BMI after 10 weeks. All results and significance values are presented in Table 3. No participants reported any injuries during the 10-week study.

**Table 3:** Fitness and performance changes for both groups using intention to treat analysis

Variable	Mean Change (M ± SD) TF20 (n = 7) CG (*n* = 6)		Within Groups Comparison^a^ TF20 CG		Between Groups Comparison^b^
Weight (kg)	−0.39 ± 2.9	− 0.55 ± 1.0	1.000	0.109	0.153
% Fat	**−2.09 ± 1.1**	− 1.52 ± 1.7	0.018*	0.109	0.063
Fat Mass (FM, kg)	**−1.93 ± 1.2**	− 1.42 ± 1.7	0.018*	0.109	0.053
Lean Mass (FFM, kg)	**5.19 ± 4.0**	0.87 ± 1.7	0.028*	0.109	0.199
BMI (kg/m^2^)	**−0.03 ± 0.9**	**− 0.37 ± 0.4**	0.933	0.109	**0.046** ^**Ɨ**^
Grip Strength (adj.)	**0.08 ± 0.06**	0.03 ± 0.05	0.018*	0.109	**0.038** ^**Ɨ**^
Sit & Reach (cm)	2.96 ± 3.0	1.21 ± 3.4	0.063	0.593	0.086
Vertical Jump (cm)	−1.02 ± 2.7	−0.85 ± 3.5	0.684	0.655	1.000
Push-Ups	4.43 ± 11.0	0.67 ± 2.1	0.249	0.414	0.830
Curl-Ups	6.86 ± 22.0	10.5 ± 16.2	0.176	0.180	0.315
Agility (sec)	**−0.57 ± 0.6**	0.04 ± 0.1	0.028*	0.180	0.775
VO_2max_ (ml/kg/min)	**2.47 ± 1.1**	1.99 ± 2.2	0.028*	0.109	0.685

*%FAT* % body fat, *BMI* Body Mass Index, *VO2max* estimated VO2max

*Statistically significant (*p* ≤ 0.05) within group change. ƗStatistically significant (*p* ≤ 0.05) difference between groups. ^a^Mann-Whitney U. ^b^Wilcoxon Signed Rank

All entried in boldface are statistically significant (*p* > 0.05); this is noted in the table notes

### Within group changes on fitness measures

All variables were examined from pre- to post-intervention. For within-groups comparisons, we used the ITT model with baseline observations carried forward for those without follow-up scores. The Wilcoxon Signed Rank Test revealed no significant changes within the CG (Table 3). In TF20 group, the Wilcoxon Signed Rank test revealed statistically significant within-group improvements in body fat percentage, fat mass (kg), lean mass (kg), grip strength, agility time, and estimated VO_2max_ from baseline to follow-up (see Table 3).

McNemar’s Test revealed that neither group showed statistically significant differences in CPAT pass rates from pre- to post-intervention. The average pass rate for CG at baseline was 60% and remained 60% post-intervention under the ITT assumption of no change. The average pass rate for TF20 group at baseline was 40% and improved to 86% after the intervention. The individual pass/fail rates and times for the CPAT are listed in Table 4.

**Table 4:** Candidate Physical Ability Test Pass/Fail Scores and Times at Baseline and Posttest

Participant	Baseline		Posttest	
	Pass/Fail	Time	Pass/Fail	Time
CG-1	P	8:57	P	8:21
CG-2	P	8:13	P	7:46
CG-3	P	9:39	P	8:31
CG-4	F	2:15^a^	F	2:15^a^
CG-5	–	–	–	–
CG-6	F	21:58	F	21:58
TF20–1	P	10:04	P	9:07
TF20–2	F	11:54	F	2:34^a^
TF20–3	P	8:36	P	8:19
TF20–4	F	11:01	P	9:33
TF20–5	–	–	P	9:20
TF20–6	–	–	P	7:36
TF20–7	F	12:32	P	10:20

*P* Pass (i.e., completed in 10:20 or less), *F* Fail, indicates the participant did not complete this measure. ^a^Participant chose not to complete the entire CPAT course after failing on the first event

Though the CPAT is a timed test with a time limit of 10 min and 20 s, we encouraged the participants to go through the entire course and recorded their overall time to completion. This permitted us to observe improvements in time to completion, even if the candidate did not improve from a failing to a passing time. We did have one participant who, though he had a failing time, completed the test at baseline. However, at post-intervention testing, the candidate chose not to complete the entire CPAT course after failing on the first event. Two participants were unable to take the CPAT test at baseline due to scheduling conflicts; their post-intervention scores are listed.

### Feasibility analysis

To examine the strengths and weaknesses of TF20 intervention, a feasibility analysis was conducted. Participants were asked about adherence, if they found the workouts challenging, any issues they faced, and suggestions for future exercise interventions offered at the Fire Academy. TF20 participants completed on average 75% of the assigned workouts, accounting for all workouts participants completed, including those the online system did not log. Participants noted experiencing difficulties logging into the application (app) on their mobile device. They reported that sometimes the app would correctly reflect that they had completed and logged a workout but at other times the system did not save their progress after they logged out of the application.

Participants also noted it was difficult to maintain a workout regimen during the intense 12-credit hour Fire Academy. Although the exercise program was designed to be flexible so participants could complete workouts virtually anywhere, numerous participants noted they had a difficult time completing the workouts without access to a gym or fire station. Multiple participants noted that having a structured exercise program with a trained individual to lead them through workouts may be more beneficial than participants trying to work out on their own.

## Discussion

The purpose of this pilot investigation was to assess performance outcomes, acceptability, and feasibility of an innovative firefighter fitness and wellness program (TF20) on firefighter recruits’ health, fitness, and performance. We hypothesized that TF20 participants would demonstrate greater improvements in fire ground performance, body composition, and strength than the CG participants. This pilot study examined the program’s performance and acceptability in “real world conditions” to determine if TF20 training program was relevant for firefighter recruits as they progressed through a fire academy.

It is interesting to note that prior to participation in the training program, all firefighter recruits in this study demonstrated aerobic capacity levels below that which is deemed essential for safe and effective fire ground operations [29, 33]. Previous reports have stated that the most demanding firefighter tasks, which also were the most commonly encountered, demanded a mean VO_2_ of 41.5 ml/kg/min (range of 36.6–44.0 ml/kg/min) [33]. The WFI recommends maximal oxygen uptake of at least 42 ml/kg/min to meet the aerobic demands of the job [29]. Prior to training, fire recruits in this study possessed an estimated average VO_2max_ of 38.9 ml/kg/min (CG mean = 39.8 ml/kg/min, TF20 mean = 38.5 ml/kg/min). After the 10-week intervention the CG had a mean estimated VO_2max_ of 43.8 ml/kg/min and TF20 had a mean estimated VO_2max_ of 41.1 ml/kg/min.

The American College of Sports Medicine (ACSM) classifies VO_2max_ values between 38.0–41.0 ml/kg/min in the “poor” range for males between the ages of 20–29 years [27]. In line with previous firefighter research, individuals with decreased comprehensive fitness levels (i.e., VO_2max_) are at an increased risk of injury, clearly showing a need to better prepare all firefighters for the physical demands of firefighting [9, 15, 27]. Our study findings also support the current literature that suggests the general public is not as fit as they should be, thereby effectively lessening those qualified for physically demanding jobs such as firefighting and other tactical occupations (e.g., military and police).

At baseline, 67% of the CG and 80% of TF20 group reported meeting moderate/vigorous PAG [34]. At the end of the 10-week study, all nine of the participants that took the follow-up questionnaire (TF20 = 6, CG = 3) reported meeting or exceeding the aerobic portion of the PAG. Four TF20 participants met the full PAG at baseline and at follow-up. These findings may be due, in part, to the physically demanding nature of the Fire Academy (e.g., climbing ladders, navigating through dark search quarters). Fire ground activities may increase aerobic activity so the nature of the Fire Academy itself, may be the reason for the increase in reporting meeting aerobic guidelines.

The CPAT was used as a critical occupational performance measure since it (or an equivalent test) is required for employment by most fire departments across the country. TF20 participants showed marked improvement on their CPAT performance, even with a small sample size. It is possible that improvement on the CPAT was due to familiarization with fire ground-related tasks in the fire academy and not the intervention itself; the only two participants who failed the test at baseline and passed at post-intervention were in TF20 group. However, occupationally-relevant physical agility tests are related to several fitness and body composition parameters [16]. Thus, recruits may learn the basic firefighter skills while in an academy, yet lack the physical fitness to pass the CPAT. This shows the importance of implementing a culture of fitness early in a firefighter’s career. If fitness programs can be implemented at the fire academy level, physical fitness training can become habitual for young firefighters.

Results from this study show promise for utilizing TF20 as part of a fire academy-specific training program to begin instilling the importance of a fit fire service at the start of their careers. Findings from this and other fitness intervention studies [6, 35, 36] support the development and implementation of physical training programs for firefighters. Future research should examine TF20 program in a larger population to determine if it significantly impacts the fitness and performance of firefighter recruits as well as career and volunteer firefighters.

### Limitations

This study was designed primarily as a pilot study, with the goals of testing the feasibility of implementing the intervention, carrying out the assessment protocol, and deriving parameter estimates from the primary outcomes and attrition. Given the pilot nature of this study, there are several limitations that should be noted including the small initial sample size (*n* = 13). The fire academy from which we recruited only had a very small initial group from which we could recruit (*n* = 24), thus limiting our starting sample size.

Second, because this was a pilot study with minimal funding, we were limited in the amount of contact we could provide participants and were unable to offer any incentives, unlike other exercise studies. This resulted in a third study limitation, which was high and differential attrition in the CG (50%) vs. TF20 group (0%). This high CG attrition and the small initial sample led to an even smaller post-treatment sample size for the completers’ analysis (and further limited power to detect group differences in outcomes), as well as negatively impacting our ability to address missing data using common imputation methods for ITT analysis as suggested in the CONSORT Guidelines for clinical trials.

Another limitation was equipment availability. For example, because the site of data collection was two hours away from our laboratory, we were unable to complete maximal aerobic capacity tests for each participant. Instead, based on recommendations from the WFI, we used a StepMill submaximal graded exercise test to estimate VO2max. Though there are errors associated with submaximal exercise tests, a recent review article found that submaximal step tests provide a simple, effective, and valid method of submaximally assessing VO2max [37].

While weighted vests required for use in the CPAT do not elicit the same physiologic burden as firefighting in full turnout gear, the weighted vest, as well as the other equipment used in the CPAT, were designed to provide the highest level of consistency, safety, and validity in measuring each participant’s physical ability and occupational readiness. In addition, they represent the recommended “gold standard” set by the IAFF/IAFC WFI for recruit firefighter fitness testing [24, 29]. Load carriage decreases exercise tolerance, capacity, and efficiency, although the shape (i.e., a weighted vest vs. protective clothing) and the placement of the load do have an impact on physical performance [38, 39].

Additionally, a longer intervention (> 10 weeks) may be beneficial as the current 10-week intervention did not show as many improvements as some 16-week interventions [1, 36]. However, fitness improvements were found for military personnel after only 8-weeks of trainer-led circuit-based training [8], which was a suggestion from our study participants.

## Conclusion

This study is the first to systematically document the effects of TF20 and is one of few to examine firefighter recruits specifically. This investigation provides promising results for the feasibility, acceptability, and potential efficacy of high-intensity training programs designed for the fire service. It also provides useful information that will aid in the design and implementation of a larger randomized controlled trial and provides alternative guidance for exercise prescription specifically for firefighters. Further investigation is necessary with a larger sample population to examine different types of physical training and its effects on the firefighter population, specifically firefighter recruits. Future studies should also examine the impact of load carriage on firefighter performance and examine the difference between weighted vests and full firefighter turnout gear on physiologic performance.

## Abbreviations

ACSM: American College of Sports Medicine
BF%: Body Fat Percentage
BIA: Bioelectrical Impedance Analysis
BMI: Body Mass Index
CDC: Centers for Disease Control and Prevention
CG: Control Group
CPAT: Candidate Physical Ability Test
CPR: Cardiopulmonary Resuscitation
CSCS: Certified Strength and Conditioning Specialist
CVD: Cardiovascular Disease
DEXA: Dual X-ray absorptiometry analysis
EMT: Emergency Medical Technician
FFM: Fat-free Mass
FM: Fat Mass
GS: Grip Strength
IAFC: International Association of Fire Chiefs
IAFF: International Association of Firefighters
IPAQ: International Physical Activity Questionnaire
IRB: Institutional Review Board
KSU: Kansas State University
LODD: Line of Duty Death
NFPA: National Fire Protection Association
PAG: Physical Activity Guidelines
PU: Push-ups
SCBA: Self-contained Breathing Apparatus
SFGT: Simulated Fire Ground Test
TF20: The First Twenty Tactical High Performance Program
TSAC-F: Tactical Strength and Conditioning Facilitator
USDHHS: US Department of Health and Human Services
VO_2max_: Maximal aerobic capacity
WFI: Wellness Fitness Initiative
